# Safety and efficacy of rituximab as first- and second line treatment in multiple sclerosis – A cohort study

**DOI:** 10.1177/2055217320973049

**Published:** 2021-01-31

**Authors:** Hilde Marie Torgauten, Kjell-Morten Myhr, Stig Wergeland, Lars Bø, Jan H Aarseth, Øivind Torkildsen

**Affiliations:** Neuro-SysMed, Department of Neurology, Haukeland University Hospital, Bergen, Norway; Department of Clinical Medicine, University of Bergen, Bergen, Norway; Norwegian Multiple Sclerosis Registry, Department of Neurology, 60498 Haukeland University Hospital, Bergen, Norway; Department of Clinical Medicine, University of Bergen, Bergen, Norway; Norwegian Multiple Sclerosis Competence Centre, Department of Neurology, 60498 Haukeland University Hospital, Bergen, Norway; Norwegian Multiple Sclerosis Registry, Department of Neurology, 60498 Haukeland University Hospital, Bergen, Norway; Neuro-SysMed, Department of Neurology, 60498 Haukeland University Hospital, Bergen, Norway; Department of Clinical Medicine, University of Bergen, Bergen, Norway

**Keywords:** Rituximab, multiple sclerosis, treatment response, efficacy, adverse effects, disease modifying therapies

## Abstract

**Background:**

Rituximab is increasingly used as off-label therapy in multiple sclerosis (MS). More data are needed on safety and efficacy of rituximab, particularly in cohorts of de novo patients and patients in early therapy escalation.

**Objective:**

To investigate the safety and efficacy of off-label treatment with rituximab in an MS-cohort of predominantly de novo patients or as therapy escalation.

**Methods:**

We retrieved safety and efficacy data from the Norwegian MS-registry and biobank for all MS-patients treated with rituximab at Haukeland University Hospital, Bergen, Norway, during a four year period.

**Results:**

In the 365 MS-patients (320 relapsing-remitting MS (RRMS), 23 secondary progressive MS (SPMS), and 22 primary progressive MS (PPMS)), the overall annualized relapse rate (ARR) was 0.03 and annualized drug discontinuation rate (ADDR) was 0.05. NEDA-3 was achived in 79% of patients with available data (n=351). Sixty-one patients experienced infusion-related adverse events of which two were serious (CTCAE grade 3–4). Eighteen patients experienced serious non-infusion related adverse events, of which 16 were infections. Infections (n = 34; 9.3%, CTCAE grade 2-5), hypogammaglobulinemia (n = 19, 5.2%) and neutropenia (n = 16; 4.4%) were the most common non-infusion-related adverse events.

**Conclusion:**

Rituximab was a safe and highly efficient disease modifying therapy in this cohort of MS-patients; however, infections and neutropenia need to be monitored.

## Introduction

Increasing evidence suggests that B-cell depletion is a safe and highly effective therapy option in multiple sclerosis (MS).^1^ More than a decade ago, the first randomized, controlled phase II trial of an anti-CD20-antibody (rituximab) demonstrated high efficacy in MS.^2^ Rituximab was not included in further development programs in relapsing-remitting MS (RRMS), but the humanized monoclonal anti-CD20-antibody ocrelizumab has recently proved effective in phase II/III trials, both in RRMS and primary progressive MS (PPMS).^3,4^ Emerging real world data indicate a comparable and possibly superior effect and tolerability of rituximab compared to other standard MS-treatments.^5–12^

Rituximab has to a large extent been used as a last-resort MS-treatment, but high efficacy treatments are now more frequently used at an early disease stage. At Haukeland University Hospital, Western Norway, rituximab has been the preferred therapeutic option for highly active MS in treatment naive patients since 2016, as well as an equivalent option for therapy switch or escalation in MS. Hence, rituximab has become the most commonly used MS-immunomodulatory therapy at our hospital. Based on the experience from this treatment approach, we conducted a retrospective cohort study to describe safety and efficacy of rituximab therapy in MS patients at Haukeland University Hospital during 2015–2019.

## Material and methods

This study was a single-centre, retrospective cohort study of all MS-patients who initiated rituximab therapy from 01.01.15 until 01.01.19 and consented to registration in the National MS registry at the Department of Neurology, Haukeland University Hospital, Bergen, Norway. Patients were identified through the Norwegian MS registry and cross-checked with the Department's list of patients treated with rituximab. From 2016 rituximab became the drug of choice for highly active disease both in therapy escalation and in treatment naïve patients, i.e. patients with two or more of the following characteristics: young age, severe function loss or extensive MRI-lesions. Observation time was set from the date of treatment initiation (at latest 01.01.19) until 01.07.19 if rituximab was not discontinued. If patients discontinued rituximab, observation time was set until six months after the latest rituximab infusion, or the date of initiation of a new disease modifying therapy (DMT).

### Study variables

Data were retrieved from the Norwegian MS Registry and biobank and included age, sex, date of disease onset, number of relapses in total and during the last two years prior to rituximab therapy, previous treatment, and reason for initiation or switch to rituximab. Efficacy recording included date of any relapse or new magnetic resonance imaging (MRI) T1 gadolinium henhancing (T1Gd+) lesion, or new T2 lesion, and change in Kurtzke Expanded Disability Status Scale (EDSS) from baseline to latest observation, at least six months from rituximab initiation. Safety recording included date and reason for rituximab discontinuation, and adverse events.

### Treatment regimen and monitoring

The majority of patients received a single infusion of rituximab 1000 mg intravenously (i.v.) at initiation, followed by 500 mg i.v. every 6 months. Patients who started rituximab treatment between 01.01.2015-31.12.2016 normally received a dose of total 2000 mg i.v. at initiation (given as two single infusions of 1000 mg i.v. within an interval of two weeks) followed by 500 mg i.v. every six months (Table 1).

**Table 1.** Protocol of rituximab dosing and administration.

Substance	Administration route	Dosage
Rituximab	i.v.	1000 mg at 0 days, 500 mg every 6 months (Patients initiating treatment between 01.01.2015 and 31.12.2016: 1000 mg at 0 days, 1000 mg at 14 days, 500 mg every 6 months.)
Paracetamol	p.o.	Premedication with an antipyretic (paracetamol 1000 mg tablet) approximately 30-60 minutes prior to each infusion.
Cetirizine	p.o.	Premedication with an antihistamine (cetirizine 10 mg tablet) approximately 30-60 minutes prior to each infusion;
Methylprednisolone	i.v.	Premedication with 125 mg intravenous methylprednisolone approximately 30 minutes prior to each infusion
**In case of infusion reactions:**		
Cetirizine	p.o.	Cetirizine 10 mg tablet

MRI scans were performed on 1.5 T or 3 T scanners. The most recent MRI before rituximab initiation was defined as the baseline MRI scan. A re-baseline scan was planned 6 months after rituximab treatment initiation, followed by a new MRI scan at 12 months, and further every 12 months, or if clinically indicated. Brain MRI scan was performed routinely, and a spinal MRI scan was performed if clinically indicated. An EDSS was planned as part of clinical evaluation in the outpatient clinic at latest six months after rituximab initiation, and at regular follow-up every 6–12 months. Laboratory tests including hemoglobin (Hb), white blood cells with differential count, platelet count, ESR (erythrocyte sedimentation rate), CRP (C-reactive protein), liver function parameters (GGT (Gamma-Glutamyl Transferase), ALT (alanine transaminase, AST (Aspartate transaminase), ALP (alkaline phosphatase)), kidney function (creatinine), IgG and IgM (immunoglobuline G and M) were performed every 6 months.

### Study outcomes

Efficacy outcomes included (i) annual relapse rate, (ii) number of patients experiencing new MRI-disease activity during observation, defined as new T2 or T1Gd+ lesions, and (iii) disability progression, measured as change in EDSS (0.5 points or more), and (iv) proportion of patients with no evidence of disease activity (NEDA-3). NEDA-3 was defined as a composite score comprising absence of clinical relapses and disability progression, in addition to no new MRI disease activity (new T1Gd+ or new/enlarging T2-lesions) on MRI examinations for the given period. Treatment outcomes were evaluated for the period before the re-baseline MRI (i.e. the first six months of therapy) and after the re-baseline MRI (i.e. >six months of therapy). Safety outcomes included all reported adverse events, including infusion-related adverse events, except mild infections (Common Terminology Criteria for adverse events (CTCAE) grade 1), which were not included due to suspected incomplete registration in the registry and patient records.

### Statistical methods

Survival (relapse-free survival, MRI event-free survival, progression free survival) was estimated with Kaplan–Meier survival curves. Analyses were performed in SPSS version 24 (IBM Corp., Armonk, NY). Descriptive data were described as means, medians and percentages.

## Approval and patient consent

All data were retrieved from the Norwegian MS Registry and biobank, where participants have given a prospective informed consent. The study was approved of by Regional Committee for Medical Research Ethics, Western Norway (REK no 87388).

## Results

### Patient characteristics

A total of 365 MS-patients (320 RRMS, 23 SPMS, 22 PPMS) who initiated disease modifying treatment in Haukeland University Hospital, Department of neurology with rituximab during the predefined study period, and consented to registration in the Norwegian MS registry, were included. One patient (n=1, RRMS) was identified through the Department's list of patients but had refused consent to the registry and was not included. Table 2 presents baseline characteristics. Mean (SD) age was 42.3 (±12.2) years and median (range) disease duration was 5.3 (± 7) years at rituximab initiation. Mean observation time was 610 days (±277).

**Table 2.** Demographic and clinical characteristics of MS-patients at initiation of rituximab therapy.

	Subgroups				
	RRMS(n = 320, 87.7%)	SPMS(n = 23, 6.3%)	PPMS(n = 22, 6.0%)		Total(n = 365, 100%)
Female, n (% of subgroup)	231 (72.1 %)	14 (60.9 %)	10 (45.5 %)		255 (69.9 %)
Age (years) at rituximab initiation, mean (SD)	40.4 (±10.9)	54.9 (±10.3)	57.3 (±12.2)		42.3 (±12.1)
Disease duration (years since diagnosis), mean (SD)	4.8 (±6.5)	15.2 (±9.1)	2.7 (±4.4)		5.3 (±7.0)
EDSS at rituximab initiation, median (range)	1.5 (0–7.5)	6 (3.0–7.5)	4 (1.0-8.0)		2.0 (0-8.0)
Number of previous DMTs, median (range)	1 (0–10)	2 (0–4)	0 (0–2)		1 (0–10)
Treatment naïve patients, n (%)	104 (32.5 %)	4 (17.4 %)	18 (81.8 %)		126 (34.5%)
Reason for switch to rituximab, n (%)					
– Newly diagnosed					104 (28.5%)
– Adverse events on other DMT					70 (19.2%)
– Treatment failure on other DMT					98 (26.8%)
– JCV positive					21 (5.8%)
– Planned pregnancy					9 (2.5%)
– Disease progression in not treated patient					38 (10.4%)
– Other					25 (6.8%)
First dose of 2000 mg, n (%)	35 (10.9 %)	6 (26.1 %)	3 (13.6 %)		44 (12.1 %)
Number of rituximab infusions, mean (SD)	3.7 (±1.5)	3.2 (±1.8)	3.2 (±1.6)		3.6 (±1.6)
Patients with any prolonged dose interval, n (%)	41 (12.8%)	1 (4.3%)	1 (4.5%)		43 (11.8%)
Cause:					
• Pregnancy/planned pregnancy (n)	19	0	0		19
• Adverse event (n)	14	0	0		14
• Intercurrent disease (n)	5	1	0		6
• Other reason (n)	3	0	1		4
Observation time (days), mean (SD)	620.0 (±274)	560.0 (±312)	516.5 (±271)		610.0 (±277)
Observation time (years), number of patients (n)					
<1 year	61	5	8		74
≥1 year	259	17	15		291
≥2 years	120	5	8		133
Baseline MRI before rituximab initiation, n (%) Any re-baseline or later MRI available, n (%)	319 (99.6%) 313 (97.8%)	23 (100%) 23 (100%)	22 (100%) 22 (100%)	364 (99.7%) 358 (98%)	
Annualized relapse rate (ARR) last 2 years before study initiation	0.52	0.34	0.12	0.55	

About one third of the patients (n = 126; 34.5%) were treatment naïve, either newly diagnosed (within the last 2 months (n = 104, 28.5%)) or never received DMT (n = 22, 6.0%). The rest were switched from different interferon beta preparations (n = 12; 3.3%), glatiramer acetate (n = 11; 3.0%), teriflunomide (n = 49; 13.4%), dimethyl fumarate (n = 47; 12.9%), natalizumab (n = 42; 11.5%), alemtuzumab (n = 6; 1.6%), fingolimod (n = 48; 13.2%), other (mycophenolate, n = 2, 0.5%) or haematopoetic stem cell transplantation (HSCT) (n = 2; 0.5%), or did at the moment not receive DMT (n = 20, 5.5%). Treatment failure was the most common reason for swithching from another DMT (n = 98; 26.8%).In patients receiving no treatment (n = 42), disease progression was the most common reason for initiating rituximab (n = 38; 10.4%).

### Treatment response

Relapse rate, frequency of new MRI lesions, EDSS change and drug discontinuation rate were calculated and are all displayed in Table 3.

Observation time for patients in the study was <12 months in 74 patients, and ≥12 months in 291 patients. In 133 patients, observation time was ≥24 months.

**Table 3.** Treatment response and discontinuation in MS patients receiving rituximab.

	Subgroups			Total (365)
	RRMS (320)	SPMS (23)	PPMS (22)	
Relapses during observation, n	15	1	0	16
– Relapses occuring after 6 months, n	8	1	0	9
Annual relapse rate (ARR)	0.03	0.03	0	0.03
– ARR calculated for observationtime and relapses occuring after 6 months	0.02	0.04	0	0.02
Patients with any new or enhanced MRI- lesions, n	52	4	2	58
– Patients with new or enhanced MRI- lesions ≥6 months	13	3	1	17
EDSS change, median (range)	0 (−3.0 −2.5)	0 (0.0 – 2.0)	0 (−1,0 – 1.5)	0 (--3.0 – 2.5)
– Improved EDSS, n (%)	18 (5.7%)	0	1 (4.5%)	19 (5.4%)
– Stable EDSS, n, %	282 (89.8%)	16 (72.7%)	18 (81.8%)	316 (89.8%)
– Worsened EDSS, n, %	10 (3.2%)	6 (27.3%)	1 (4.5%)	17 (4.6%)
NEDA-3 at end of observation, n (%)	80.3%	57.1%	81.0%	79.0%
Drug discontinuation, n (%) Annual drug discontinuation rate (ADDR)	23 (7.2%) 0.04	7 (30.4%) 0.20	3 (13.6%) 0.10	33 (9%) 0.05
Reason for discontinuation, n (% of total)				
– Treatment failure	1	1	0	2 (0.5%)
– Adverse events	13	3	2	18 (4.9%)
– Patient's request of HSCT	4	2	0	6 (1.6%)
– Patient's request of discontinuation	4	1	1	6 (1.6%)
– Other	1	0	0	1 (0.3%)

#### Clinical relapses

The overall annualized relapse rate (ARR) in this cohort was 0.03 (RRMS 0.03, SPMS 0.03, PPMS 0). During the first six months of treatment, fourteen patients experienced a relapse of which two had a further relapse. After six months of treatment or more, seven patients experienced a relapse and two had a further relapse. When excluding observation time and early relapses <6 months of treatment, with the intention of looking at treatment effect after 6 months, ARR was calculated to 0.02. In two patients, repeated relapses or a relapse after six months of treatment was the reason for drug discontinuation. 

In patients switching to rituximab after HSCT (n=2), new relapses occurred in one.

#### Subgroup analyses

ARR for the subgroup receiving an initial dosage of 2000 mg (1000 mg + 1000 mg) was 0.05 (n = 44), and 0.02 for an initial dosage of 1000 mg (n = 321).

ARR in the subgroup of newly diagnosed patients was 0.02 (n = 104), and 0.03 in other patients (n = 219) during study observation time.

#### MRI lesions

One or more control MRIs during rituximab treatment were available for 358 patients (98%). New or enhanced MRI-lesions occurred in 58 patients (n = 52 in RRMS, n = 4 in SPMS, n = 2 in PPMS). When excluding MRI events before six months of treatment, 17 patients experienced new or enhanced MRI lesions. In nine of these, no early MRI scan (<6 months) after rituximab initiation was available.

Two patients discontinued rituximab due to treatment failure; both experienced relapses and more than one MRI event. 

#### Disability progression

**Figure 1.**
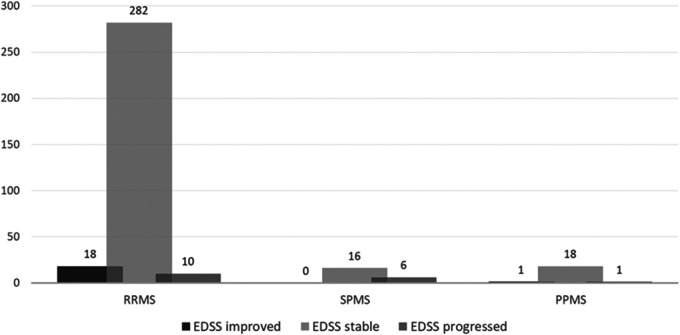
Change in EDSS during rituximab treatment in the 361 patients with at least two EDSS-evaluations.

EDSS was available at baseline and after at least 6 months of treatment for 352 patients (96%). In 316 (89.8%) patients EDSS was unchanged between their first and last available score. In RRMS patients, EDSS improved in 18 (5.7%), and worsened in ten (3.2%) patients. In the progressive disease subgroup, EDSS improved in one (3.4%) and worsened in seven (16.7%) patients. In progressive disease courses, observation time was less than a year in 13 patients, 1-2 years in 32 patients, and ≥ 2 years in 13 patients. Figure 1 displays change in EDSS related to MS subgroup.

#### NEDA (no evidence of disease activity)

NEDA- 3 was available for calculation in 351 patients. 279 of 351 (79%) patients with available data fulfilled the criteria for NEDA-3 (no new MRI lesions, no EDSS progression or clinical relapses) throughout their observation time in this study.

#### Drug survival

**Figure 2.**
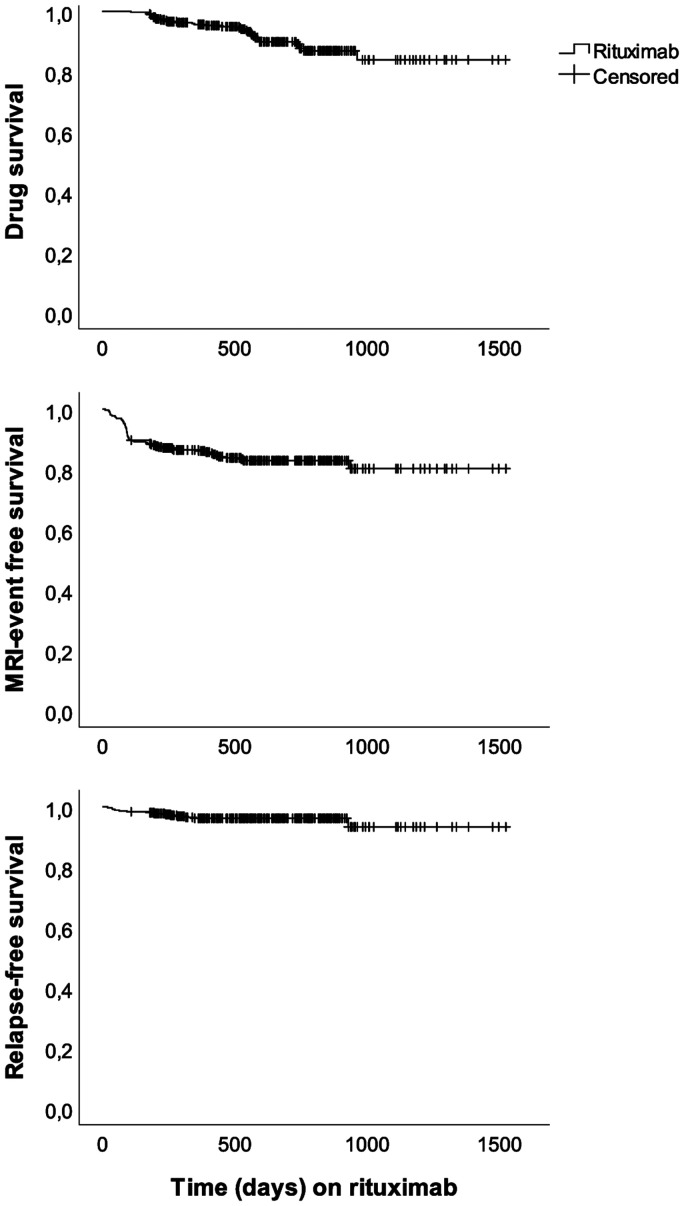
Disease activity free (MRI and relapses) patients and treatment continuation in 355 MS-patients receiving rituximab treatment.

Treatment was discontinued in 33 (9%) patients, resulting in an annual drug discontinuation rate (ADDR) of 0.05 (RRMS 0.04, SPMS 0.20, PPMS 0.10). The most frequent reason for discontinuation was adverse events (n = 18, 4.9%, CTCAE grade 1–2 in 14 cases, grade 3–4 in 4 cases). In two patients (0.5%), discontinuation was due to treatment failure (new MRI-lesions and clinical relapses). Six patients (1.1%) requested rituximab discontinuation in order to receive HSCT abroad. Figure 2 demonstrates MRI event-free survival, relapse-free survival and drug survival on rituximab during observation time.

#### Safety

Safety data results are displayed in Table 4. 118 patients (32.3%) experienced a total of 156 registered adverse events.

**Table 4.** Safety and adverse events in MS-patients receiving rituximab therapy.

	Subgroups			
	RRMS (320)	SPMS (23)	PPMS (22)	Total (365)
Patients who experienced any adverse event, n (%)	105 (32.8%)	8 (34.8%)	5 (22.7%)	118 (32.3%)
Number of adverse events, n	142	6	8	156
Patients who experienced any infusion-related adverse event (CTCAE grade 1-5), n (%)	56 (17.5%)	2 (8.7%)	3 (13.6%)	61 (16.7%)
– Serious infusion-related events (CTCAE grade 3–4), n (%)	2 (0.6%)	0	0	2 (0.5%)
Patients who experienced any serious non-infusion-related adverse events (CTCAE grade 3–4), n (%)	13 (4.1%)	4 (17.0%)	1 (4.5%)	18 (4.9%)
– Serious infections	11 (3.4%)	4 (17.0%)	1 (4.5%	16 (4.4%)
– Other serious non-infusion related adverse events (ventricular extrasystoles, tinnitus)	2 (0.5%)	0	0	2 (0.5%)
Patients registered with any infection (CTCAE grade 2–5), n (%)	28 (8.7%)	4 (17.4%)	2 (9.1%)	34 (9.3%)
Deaths (CTCAE grade 5), n	0	1^a^	0	1^a^
PML cases, n	0	0	0	0
Patients receiving cancer diagnosis during observation period, n	0	0	2^b^	2^b^
Patients with neutropenia, n (%)	14 (4.4%)	0	2 (9.1%)	16 (4.4%)
• Neutrophil count 0,5 – 1.0 × 10^9^/L (CTCAE grade 3)	2 (0.6%)	0	0	2 (0.5%)
• Neutrophil count <0.5 × 10^9^/L (CTCAE grade 4)	3 (0.9%)	0	1 (4.5%)	4 (1.1%)
Other laboratory parameters: Patients with hypogammaglobulinemia (IgG) of any grade , n (%)	16 (5%)	2 (8.7%)	1 (4.5%)	19 (5.2%)

^a^Suicide.

^b^Cancer coli, melanoma.

Infusion-related adverse events (CTCAE grade 1-5) were registered in 61 patients (16.7%). All were mild (CTCAE grade 1-2) except 2 cases (CTCAE grade 3): One patient reported a rash and a unilateral, mild facial paresis 30-60 min after infusion, which resolved spontaneousely within hospital admission the next 60 minutes. One patient experienced an acute generalized rash and dyspnoea during infusion, which resolved after peroral antihistamine. Both were admitted to hospital. Rituximab was continued with no further serious infusion-related adverse events.

Among non-infusion-related adverse events, 18 (5.0%) were serious (CTCAE grade 3–4). The majority (n = 16) were infections; Urosepsis (n = 3), diverticulitis (n = 2), neutropenic fever (n = 2), pneumonia (n = 3), pansinusitis (n = 1), cholecystitis (n = 1), spondylodiscitis (n = 1), neoerlichiosis (tick-borne infection) (n = 1), enteritis (n = 1) and proctitis (n = 1). The rate of infections, excluding mild cases not requiring any medical treatment or doctor's visit (CTCAE grade 1), was 17.4% in SPMS, 8.7% in RRMS and 9.1% in PPMS patients.

One patient died during the study observation time; cause of death was suicide, considered not to be related to rituximab treatment. Two patients were diagnosed with malignant disease (colon cancer, melanoma) during observation time, both were diagnosed after rituximab initiation, and both discontinued rituximab treatment. No case of progressive multifocal leukoencephalopathy (PML) was recorded.

Neutropenia (count < 1.5 × 10^9^/L) during rituximab treatment with no other known cause was recorded in 16 (4.3%) patients. 6 patients experienced moderate or severe neutropenia (count <1,0 x 10^9^/L) and expressed the following characteristics: All six patients had neutrophil count and leucocyte counts within normal range at baseline (within 2 weeks before rituximab initiation). One had experienced neutropenia earlier, which resolved. Five out of six were < 40 years of age. 1 patient had PPMS and EDSS > 6, the remaining had RRMS with EDSS 0-2. None had relevant comorbidities. Earlier treatment included HSCT (n = 1) and dimetylfumarat (n = 2), the remaining were treatment naïve (n = 3). Total number of rituximab infusions were 2–4; 1 patient had received an initial dose of 2000 mg rituximab. Two neutropene patients were admitted to hospital with febrile neutropenia, both were treated with i.v. antibiotics. One was admitted to hospital with a suspected fungal infection and received treatment with G-CSF (Granulocyte Colony Stimulating Factor). Three patients had no symptoms and neutrophile count normalized within 1-4 weeks. All patients recovered fully, but rituximab was discontinued in two cases due to recurrent neutropenia and high age, respectively.

#### Subgroup analyses

In patients with neutropenia (n = 16), 5 patients were registered with any infection as an adverse event. In patients without neutropenina (n=349), 31 patients were registered with an infection.

In patients receiving an initial dose of rituximab of 2000 mg, 3/44 (6.8%) were registered with any infection (CTCAE grade 2–5) versus 31/321 (9.7%) in patients receiving regular dose (1000 mg) at rituximab initiation, and 2/44 (4.5%) were registered with neutropenia versus 14/321 (4.4%) in patiens receiving regular dose at initiation. 6/44 (13.6%) patients who received 2000 mg rituximab at initiation were registered with hypogammaglobulinemia (low IgG) at any time during observation, compared to 13/321 (4.0%) in the group who received a regular dose at initiation. 

## Discussion

We present one of the largest cohorts of MS-patients treated with rituximab to date, utilizing real world data from the Norwegian MS Registry.

Efficacy data are in line with results from several studies of similar design^6,8,12^ including a larger cohort study of similar design and study population.^10^ Another large, recent multicentric observational study reported similar results; one difference to notice is the higher proportion of treatment naive patients in our cohort, pointing to the differences in therapeutic approach and patient selection in reported off-label MS-treatment with rituximab. Our cohort includes a relatively high proportion (28.5%) of newly diagnosed patients starting rituximab as their first choice DMT compared to many studies based on real-world data. 

The overall ARR of 0.03 was low, and so was the ARR of 0.02 calculated in >6 months of treatment, when assuming a transient initial periode of suboptimal treatment effect. The calculated ARR for patients receiving a higher starting dose of rituximab was 0.05; this subgroup of patients also started treatment earlier (2015-2016) while rituximab was not concidered a first choice treatment, and could possibly represent a subgroup with more persistent disease activity in the cohort. 

The total number of patients with new MRI lesions was lowafter initiation of rituximab, and after >6 months of treatment. A large proportion of patients starting rituximab had a recent history of disease activity (newly diagnosed patients, recent treatment failure on other DMTs, recent disease progression without ongoing therapy), which strengthens the efficacy results and indicate low inflammatory disease activity during rituximab treatment, further underlined by the low proportion of patients discontinuing treatment, and a very low proportion of patients discontinuing rituximab due to new disease activity. 

EDSS was mainly unchanged during rituximab treatment. Notably, the baseline EDSS score might have been transiently worsened in some patients at rituximab initiation due to recent relapses, which could lead to overestimation of improvement in EDSS during treatment. EDSS was recorded during regular clinical follow up and a validation of change in EDSS score was not demanded in the study design. 

We recorded a clinically significant number of patients in progressive disease courses registered with EDSS progression during rituximab treatment (7 out of 44 in SPMS and PPMS with valid EDSS scores). This is in line with previous studies reporting that disability progression may be reduced, but not completely stopped, during rituximab or ocrelizumab treatment in progressive MS disease courses.^4,14^ For progressive disease courses, the observation time was relatively short, and the number of patients (n = 45) was low, which limits further conclusions.

One of the main goals was to evaluate safety of rituximab treatment in MS. The rates of adverse events and the main categories of side effects in this cohort were comparable to those seen in populations of rheumatological and other autoimmune diseases.^15,16^ Among serious adverse events, infections was the most common, corroborating a recent report from a Swedish MS-population.^17^ Moderate and severe neutropenia (count <1.0 x 10 ^9^) was seen in 1.6% of patients in this cohort and may represent a serious risk to the patient's health. As neutrophil counts were registered only at baseline and every 6 months, we could not estimate the onset time of neutropenia or the full extent. Late onset neutropenia is a well-known side effect of rituximab treatment^18^ but has only been documented in few MS-patients treated with rituximab in retrospective studies.^19,20^ Our results indicate that neutropenia may be underreported if not monitored. 

The present study has several strengths. As a single-centre study, the treatment- and observation protocol was standardized, and all safety data and clinical records were fully available. Thus, serious adverse events should be well monitored and recorded, including laboratory monitoring and all hospitalizations, though recording of mild adverse events offer challenges. The dosing regimen in this cohort was largely homogenous, only a few patients (12.1%) received a higher initial dosing, and almost 90% of the patients receiving the regular maintenance dose regimen with no postponed doses. The study population included a relatively high proportion of treatment naive patients, contributing novel data for this subgroup. An important limitation of the present study is the retrospective, uncontrolled design, without adjustments for possibly confounding factors. The relatively short observation time, especially in progressive disease, is another limitation, and a control group would have strengthened the study.

Our data indicate that rituximab is a safe and highly effective treatment option in MS, both as first-line and escalation therapy. The extent of treatment with rituximab is still limited, most likely because it is off-label therapy, and because of the lack of phase III treatment study documentation. Systematic safety documentation is therefore important. Infections, including serious infections, seem to represent an important safety issue during rituximab therapy in MS, and the frequency of both mild and severe neutropenia could be underestimated as there is a lack of knowledge about onset time, and patients might be asymptomatic. Further studies should focus on treatment naive patients, and also how to prevent infections through improved screening or adjusted dosing or dosing intervals.
